# Clinical characteristics and outcome of critically ill children referred to a tertiary hospital in Indonesia: a prospective observational study

**DOI:** 10.1186/s12887-024-04940-7

**Published:** 2024-07-27

**Authors:** Desy Rusmawatiningtyas, Vicka Oktaria, Antonius H Pudjiadi, Firdian Makrufardi, Job. B.M. van Woensel

**Affiliations:** 1 Child Health Department, Faculty of Medicine, Public Health, and Nursing, Universitas Gadjah Mada, Yogyakarta, Indonesia; 2 Department of Biostatistics, Epidemiology and Population Health, Faculty of Medicine, Public Health, and Nursing, Universitas Gadjah Mada, Yogyakarta, Indonesia; 3 Child Health Department, Faculty of Medicine, Universitas Indonesia, Jakarta, Indonesia; 4 Department of Pediatric Intensive Care, Emma Children’s Hospital/Amsterdam University Medical Centers, University of Amsterdam, Meibergdreef 9, Amsterdam, The Netherlands

## Abstract

**Background:**

The clinical characteristics of pediatric critically ill patients who need referral to a tertiary hospital is often unknown in resource limited settings where constraints in diagnosis capacity, resources, and infrastructures are common. There is a need to increase insight in the characteristics of these patients for capacity building strengthening and appropriate resource allocation. The aim of this study was to describe the clinical characteristics and outcomes of critically ill children who are referred to a tertiary referral teaching hospital in Yogyakarta.

**Methods:**

A prospective observasional study was carried out from July 1st, 2022 -January 31st, 2023 which included all critically ill pediatric patients who were referred through the Integrated Referral System (SISRUTE) to the Pediatric Intensive Care Unit (PICU) of dr. Sardjito hospital. We excluded patients who were referred with a request for admission to the PICU, but were not admitted to the PICU due to their stable condition and lack of the need for intensive care.

**Result:**

During the study period, we received 1046 emergency referral requests for pediatric patients via SISRUTE, of those, 562 (53.7%) patients were critically ill. The reasons of PICU referral request were the need of solely intensive care 504 (89.7%), the need of multidisciplinary team care, including intensive care 57 (10.1%) and parents request 1 (0.3%). The pre-referral emergency diagnosis was shock 226 (40.3%), respiratory distress/failure 151 (26.7%), central nervous system (CNS) dysfunction 135 (24.1%), trauma 33 (5.9%) and sepsis 17 (3%). Of the 562 critically ill PICU referral requests, 473 (84.2%) requests were accepted. One hundred and eighty-one (58.7%) patients were finally admitted to the PICU, 125 (40.3%) admitted to our regular ward due to stable condition, 4 (1.3%) patients died in Emergency Departement (ED). The remaining accepted patients on request did not arrive in our facility due to various reasons. The mean (SD) response time was 9.1 (27.6) minutes. The mean (SD) transfer time was 6.45 (4.73) hours. Mean (SD) PICU and hospital length of stay was 6.7 (8.3) days and 10.2 (9.2) days respectively. PICU and hospital mortality was 24.3% and 29.7%, respectively.

**Conclusion:**

The mortality rate for critically ill pediatric patients referred to a tertiary PICU still high, with shock being the most common pre-referral emergency diagnosis. There is a discrepancy between the referring hospital’s and the referral hospital’s indication for PICU admission. The time required to reach the referral hospital is quite lengthy.

## Introduction

The under-5 mortality rate has - with approximately 24 per 1000 live births- [1, 2] decreased in Indonesia during the last decades, yet it is still far from the set targets [1]. As a low- and middle-income country (LMIC) Indonesia faces many challenges in the healthcare field particularly in addressing childhood mortality. Understaffing, inadequate funding, scarcity of healthcare expertise, insufficient resources, deficient training and education were identified as contributors to the high mortality rates among children in LMICs [3–6].

One of the main elements of primary health care is the referral system, which allows patients to obtain services at health centers with superior facilities and infrastructure [7]. The necessity of an efficient referral system for critically ill children is a determinant in child survival that is less acknowledged and understood [8–11]. Lack of pre-referral communication, burden at the referred facility (staff and infrastructure), poor transport systems contributes to an imbalance in supply and demand [8, 12].

Indonesia still face the challenge of inadequate establishment of well-organized referral systems to address the needs of critically ill children. Our country has the Integrated Referral System (SISRUTE) as a means of communication between pre-referral and referral health services. SISRUTE is an Information and Technology (IT)-based integrated health service delivery information system that is launched for improving the performance of health care facilities and for accelerating the referral process according to the patient’s medical needs and the competences necessary. SISRUTE makes use of several communication channels, including Short Message Service (SMS), Android applications, and web-based platforms. This enables prompt information exchange between pre-referral hospitals and referred health care institutions, with the goal to improve patient safety and family/patient satisfaction [13].

In our setting the attending doctors or nurses can send a referral request to multiple referral hospitals at their choice via SISRUTE website https://sisrute.kemkes.go.id. The choice of a referral hospital typically depends on the necessary medical resources, the distance from the referring hospital, and occasionally the preferences of the patient’s family. At specific referral hospitals, particularly tertiary referral hospitals, there are senior ED Nurses who have been assigned as Pre hospital Emergency Communication Centre (PECC) officers. These officers are responsible for regularly monitoring incoming referral request via SISRUTE. However, the majority of referring hospitals lack personnel of this nature, resulting of communication which is not always in real time. Phone communication is consistently available for submitting emergency referral requests to our hospital, which will be received by the PECC officers. At currently, SISRUTE does not have a specialized section to accommodate referring patients with critically ill condition, who necessitate prompt responses and decisions.

In term of transport system, we do not have a dedicated transport team for critically ill patients. The patients were stabilized and transported by the attending physician, who was primarily a general practitioner, and a nurse from the referring hospital, use their ambulance. In smaller healthcare facilities, the presence of ambulances may be restricted, leading to potential delays in the patient referral process. Limited data is available regarding the clinical profile and outcome of pediatric critically ill referral cases in Indonesia. We conducted this study to explore clinical, referral characteristics and outcome of pediatric critically ill referral cases to tertiary referral teaching hospital in Yogyakarta, Indonesia.

## Methods

### Study design and setting

A prospective observational study was conducted in the Emergency Department (ED) at Dr. Sardjito hospital, Yogyakarta. Dr Sardjito hospital is a tertiary public university-based hospital in Yogyakarta Province, Indonesia which serves approximately 4000 pediatric inpatients per year and provides ED services to an average of 2000 pediatric patients per year. The Pediatric Intensive Care Unit (PICU) is equipped with 16 medical and surgical intensive care unit beds and has approximately 900 admissions per year. Our team consists of three pediatric intensivists who manage patients in PICU. Additionally, we have general practitioners who have received specialized training in pediatrics and work in rotating shifts. The PICU at our facility adopts a semi-closed system.

### Patients and operational definition

We included all emergency referral that have requested admission to our PICU in patient aged 1 month to 18-year-old via SISRUTE from July 1st 2022 – January 31st 2023. Patients who were admitted to PICU were prospectively followed up until the discharged or the day they deceased. We define SISRUTE’s response time as the time between the referral request sent via SISRUTE by the referring hospital and the initial response from our hospital as the referral hospital. We defined the transfer time as the duration between the initial response and the arrival of the patient at our ED. We defined PICU mortality as the occurrence of patient deaths during the course of care within PICU. We defined hospital mortality as the occurrence of patient deaths during the course of care within hospital. We define early mortality as mortality that occurred ≤ 48 h after PICU admission. The Indonesian Ministry of Health current regulations adhere to the Pediatric Assessment Triangle (PAT) method of identifying and categorizing emergency diagnosis in pediatric patients. This method has been adapted for use in Indonesia, and has been widely disseminated and incorporated into medical education programs [14]. Our PICU admission standards adhere to the guidelines issued by the American Academy of Pediatrics in 2019, with necessary adjustments made to suit specific conditions in Indonesia as per the guidelines set by the Ministry of Health [15].

### Data collection tool, procedure and data quality assurance

Data collection was conducted using a structured case record form, administered by pediatric residents overseeing the ED. The initial data collection commenced immediately upon contact from the pre-referral hospital. Information gathered for referral cases including age, sex, required type of care, province of referral origin, reasons for patient referral, emergency diagnosis at the referring hospital, referral acceptance status, reasons for declining referral, and the final condition post referral request acceptance including the emergency diagnosis at OUR ED.

After admission to PICU at dr Sardjito hospital, we recorded patient demographic data, SISRUTE’s response time, transfer time, length of stay (LOS) at the PICU, overall LOS in our hospital, mortality, and early mortality. We also collected data on PELOD-2 [16] scores within the first 24 h of patients being admitted to the PICU.

### Data analysis

The results are presented in a descriptive manner, utilizing absolute numbers, percentages, medians, and interquartile ranges as suitable for the data. Data analysis was performed using Excel version 16.16.21 (Microsoft). We used the t-test for normally distributed continuous variables and the Mann-Whitney U-test for non-normally distributed continuous variables to determine differences between two populations.

We calculated the cut-off point of the PELOD 2-score in the population using the Youden’s J index. Youden’s J is the likelihood of a positive test result in subjects with the condition versus those without the condition. It is also the probability of an informed decision (as opposed to a random guess). Youden’s J index combines sensitivity and specificity into a single measure (Sensitivity + Specificity − 1) and has a value between 0 and 1. In a perfect test, Youden’s index equals 1. It is also equivalent to the vertical distance above the diagonal no discrimination (chance) line to the ROC curve for a single decision threshold [17].

### Ethical consideration

Ethic approval was obtained from the Medical and Health Research Ethic Committee (MHREC) with the Ref No: KE/FK/0907/EC/2022. Confidentiality and privacy were strictly maintained. Only the principal investigator and the research assistants can access the data. All methods throughout the study were performed in accordance with the relevant guidelines and regulations.

## Results

**Fig. 1:**
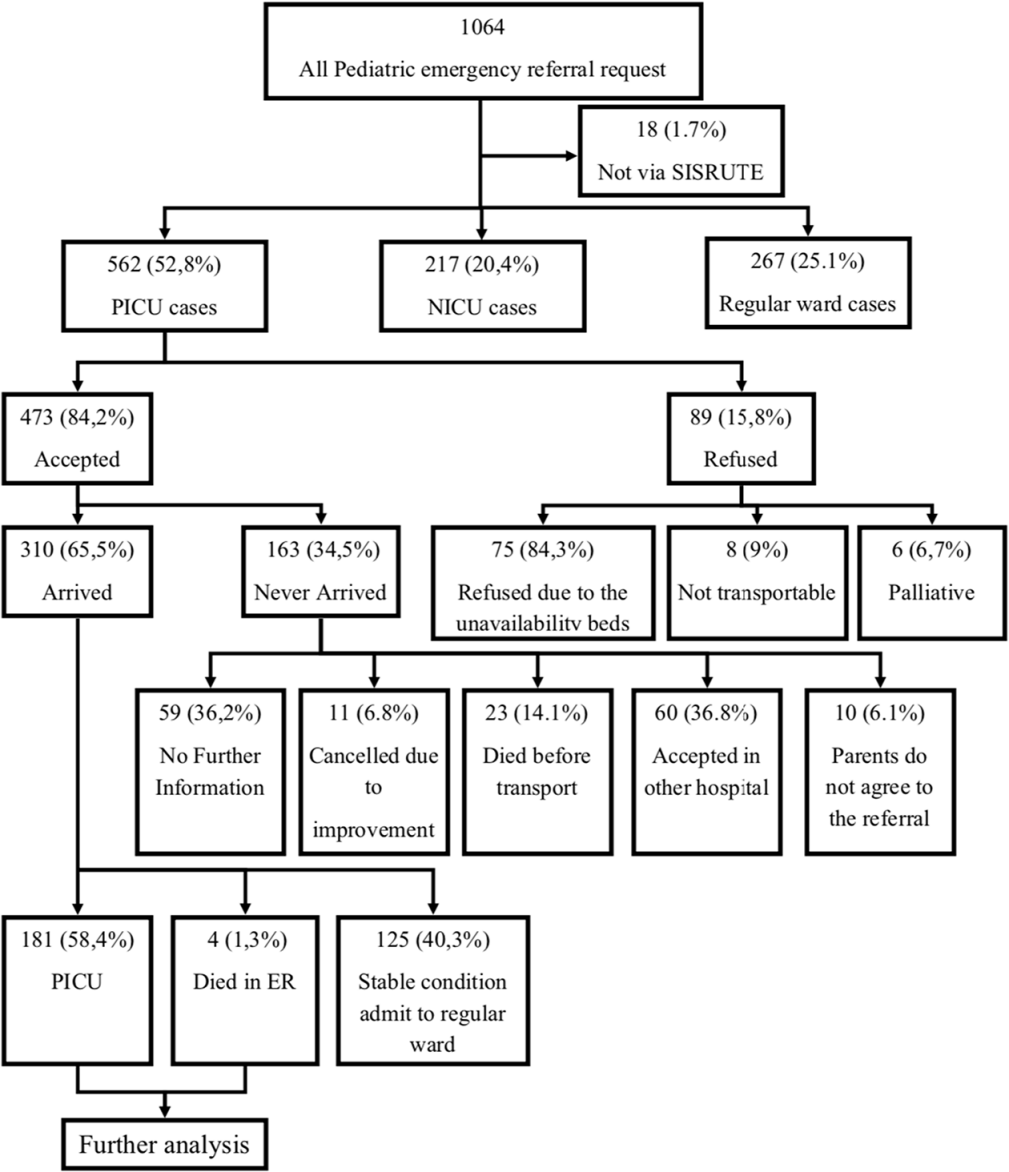
Flowchart of the study

Between July 1st 2022 – January 31st 2023, dr Sardjito hospital’s ED received 1108 pediatric and neonatal referral requests, of which 1046 were sent via SISRUTE. The 62 emergencies pediatric patient referral requests that were done directly via telephone due to errors in SISRUTE and also errors in internet connection in referring hospital are not included in the analysis. In total 562 of the 1046 SISRUTE referral requests (52.8%) were requests for PICU care, 217 (20.4%) were neonatal cases, and 267 (25.1%) were regular ward care cases. Of the 562 PICU care requests, 473 (84.2%) were accepted and 89 (15.8%) were refused predominantly due to unavailability of PICU beds (Fig. 1). Due to logistical constraints we were unable to analyze the outcome of these 89 patients. The referral origin provinces predominantly were from Yogyakarta 254 (45.2%) and Central Java 208 (37.0%) (Table 1). One case of critically ill referral request came from another island (Riau province in north Sumatra).

**Table 1:** Socio-demographic and baseline information of critically ill children referral requests at Sardjito General Hospital

Variable	Frequency (n = 562)	Percent (%)
All patient referral requests		
Aceepted,	473	84.2
Not accepted	89	15.8
Referral origin (province)		
Yogyakarta	254	45.2
Central Java	208	37.0
East Java	83	14.8
West Java	16	2.8
Other	1	0.2
Reasons for reffering the patient		
Required PICU fascility	504	89.7
Required multidiscipline team	57	10.1
Parents request	1	0.2
Pre-referral Emergency diagnosis		
Shock	226	40.3
Respiratory distress/failure	151	26.7
CNS dysfunction	135	24.1
Trauma	33	5.9
Sepsis	17	3
Sex		
Male	312	55.5
Female	250	44.5
Age		
1 month – 1 year	168	30.0
> 1–5 year	147	43.9
> 5–18 year	247	43.9

Of the 473 referral requests that initially were accepted as PICU cases, 163 patients were not transferred due to these reasons: 59 (36.2%) patients never arrived in our hospital without further information; 11 (6.8%) patients improved and their referral was cancelled; 23 (14.1%) patients died before transport; 60 (36.8%) patients were accepted to another (lower level) hospital; and the parents of 10 patients (6.1%) refused the transfer.

Of the remaining 310 patients who actually arrived at our ED, 125 (40.3%) were not critically ill and were then admitted to the regular ward. The remaining 185 (59.7%) patients were triaged as having indications for admission to the PICU. Of these, 4 (2.2%) patients died in the ED and 181 (97.8%) patients were finally admitted to the PICU.

The most common pre-referral emergency diagnosis based on PAT method was shock 226 (40.3%). The other pre-referral diagnosis was respiratory distress/failure 151 (26.7%), central nervous system (CNS) dysfunction 135 (24.1%), trauma 33 (5.9%) and sepsis 17 (3%) (Table 1). The most common diagnoses in our ED also based on PAT method were cardiorespiratory failure in 57 (30.8%) patients, respiratory distress in 43 (23.2%) patients, shock in 35 (18.9%) patients, respiratory failure in 28 (15.1%) patients and CNS dysfunction in 22 (11.9%) patients (Table 2).

**Table 2:** Characteristic of subject who admit to PICU

Characteristics	Total N = 185 (%)
SISRUTE’s responds time, mean ± SD (minute)	9.13 ± 27.61
Yogyakarta	7.97 ± 20.16
Central Java	10.88 ± 34.63
East Java	7.44 ± 9.79
West Java	2.00 ± 0.00
Transfer time, mean ± SD (hours)	6.45 ± 4.73
Yogyakarta	4.64 ± 3.65
Central Java	7.7 ± 4.95
East Java	10.54 ± 5.2
West Java	10.98 ± 3.35
Sex	
Male, n (%)	110 (59.5)
Female, n (%)	75 (40.5)
Age	
1 month – 1 year, n(%)	53 (28.6)
> 1–5 year, n (%)	46 (24.9)
> 5–18 year, n (%)	86 (46.5)
Nutritional state, n (%)	
Normal	116 (62.7)
Underweight	39 (21.1)
Severe malnutrition	12 (6.49)
Overweight	2 (1.08)
Obesity	16 (8.65)
Emergency diagnosis based on PAT^14^ in ED	
Cardiorespiratory failure	57 (30.8)
Respiratory distress	43 (23.2)
Shock	35 (18.9)
Respiratory failure	28 (15.1)
CNS dysfunction	22 (11.9)
Oxygen Therapy prior referral	
Conventional Oxygen therapy	144 ( 77.8)
Manual bag and mask	3 (1.6)
Mechanical ventilation	33 (17.8)
NIV	3 (1.6)
CPAP	1 (0.5)
HFNC	1 (0.5)
PELOD-2 Socre, mean ± SD	4.27 ± 4.17
Length of stay PICU, mean ± SD (days)	6.72 ± 8.31
Length of stay Hospital mean ± SD (days)	10.23 ± 9.24
Hospital mortality	55 (29.7)
PICU mortality	45 (24.3)
Early death	
≤ 48 hours after admission	18 (32.7)

PAT : Pediatric Assessment Triangle, CNS : Central Nervous System, NIV : Non Invasive Ventilation, CPAP : Continous Positive Airway Pressure, HFNC : High Flow Nasal Canule. SD: Standard deviation, ED: Emergency department.

**Fig. 2:**
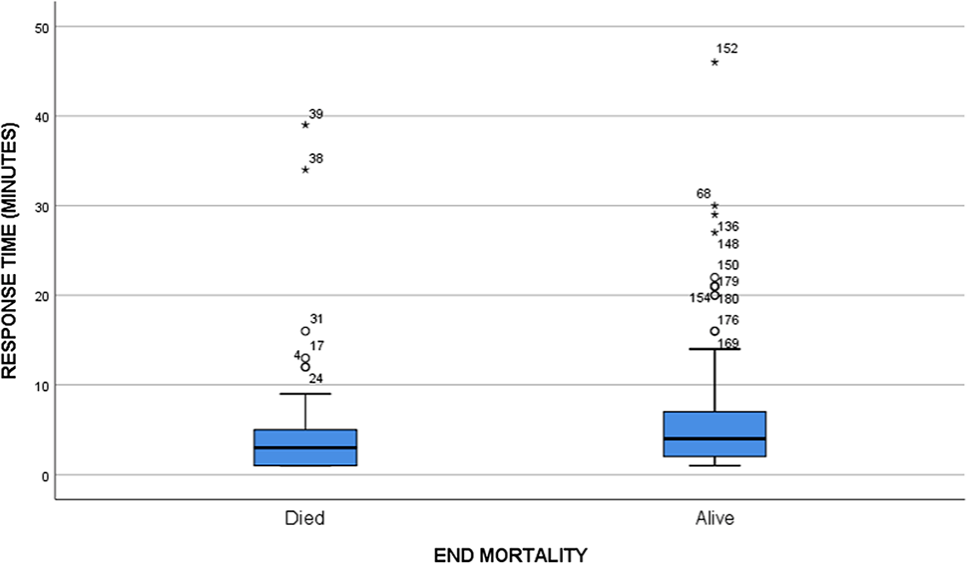
Response time (minute) between groups

**Fig. 3:**
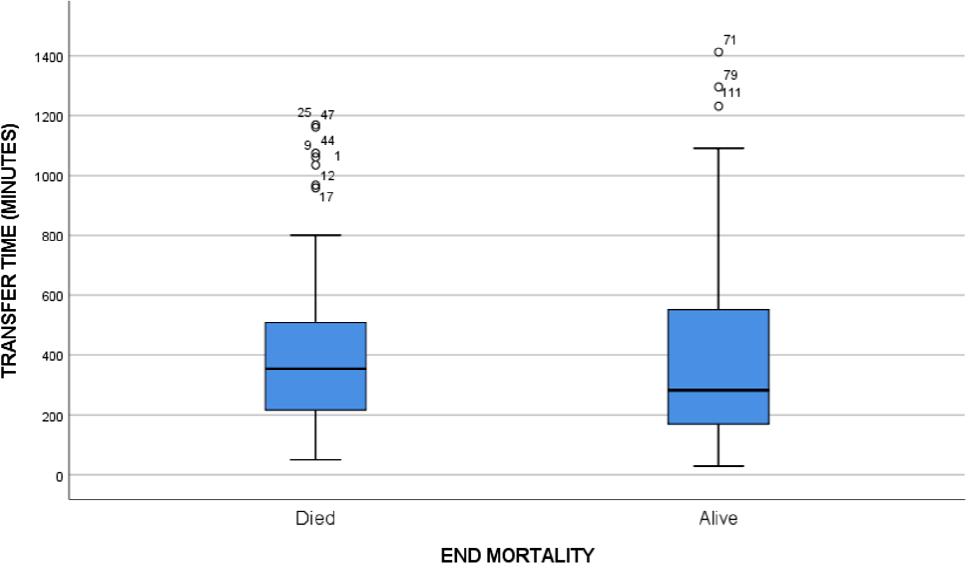
Transfer time (minute) between groups

The overall hospital mortality rate was 29.7%, of whom 32.7%. suffered from early mortality, while PICU Mortality was 24.3%. Overall mean (± SD) response time mean was 9.1 (± 27.6 ) minutes and overall mean (± SD) transfer time mean was 6.45 (± 4.73 h ) (Table 2). Both response time and transfer time were not associated with mortality rate. (Table 3). In Figs. 2 and 3, scatter plots illustrate the response time and transfer time for both deceased and survive groups.

**Table 3:** SISRUTE’s responds time and transfer time with mortality

	Hospital Mortality		*P*-value
	Deceased	Survived	
	55	130	
SISRUTE’s responds time, mean ± SD (minute)	10.46 ± 40.77	8.59 ± 20.00	0.748
Transfer time, mean ± SD (hours)	6.96 ± 4.97	6.23 ± 4.62	0.357

SD: Standard deviation

**Table 4:** Transfer time with hospital mortality

	Hospital Mortality		P value	OR (95% CI)
	Deceased	Survived		
	n; %	n; %		
Transfer time ≤ 4 h				
Yes	15 (27.3%)	56 (43.1%)	0.064	0.496 (0.249–0.985)
No	40 (72.7%)	74 (56.9%)		
Transfer time ≤ 2 h				
Yes	8 (14.5%)	16 (12.3%)	0.861	1.213 (0.486–3.025)
No	47 (85.5%)	114 (87.7%)		

OR: Odds ratio

**Table 5:** PELOD-2 score with mortality

	Hospital Mortality		*P*-value
	Deceased	Survived	
	51	130	
PELOD-2 score, mean ± SD	7.69 ± 5.08	3 ± 2.91	< 0.0001

PELOD: Pediatric Logistic Organ Dysfunction, SD: Standard deviation

**Fig. 4:**
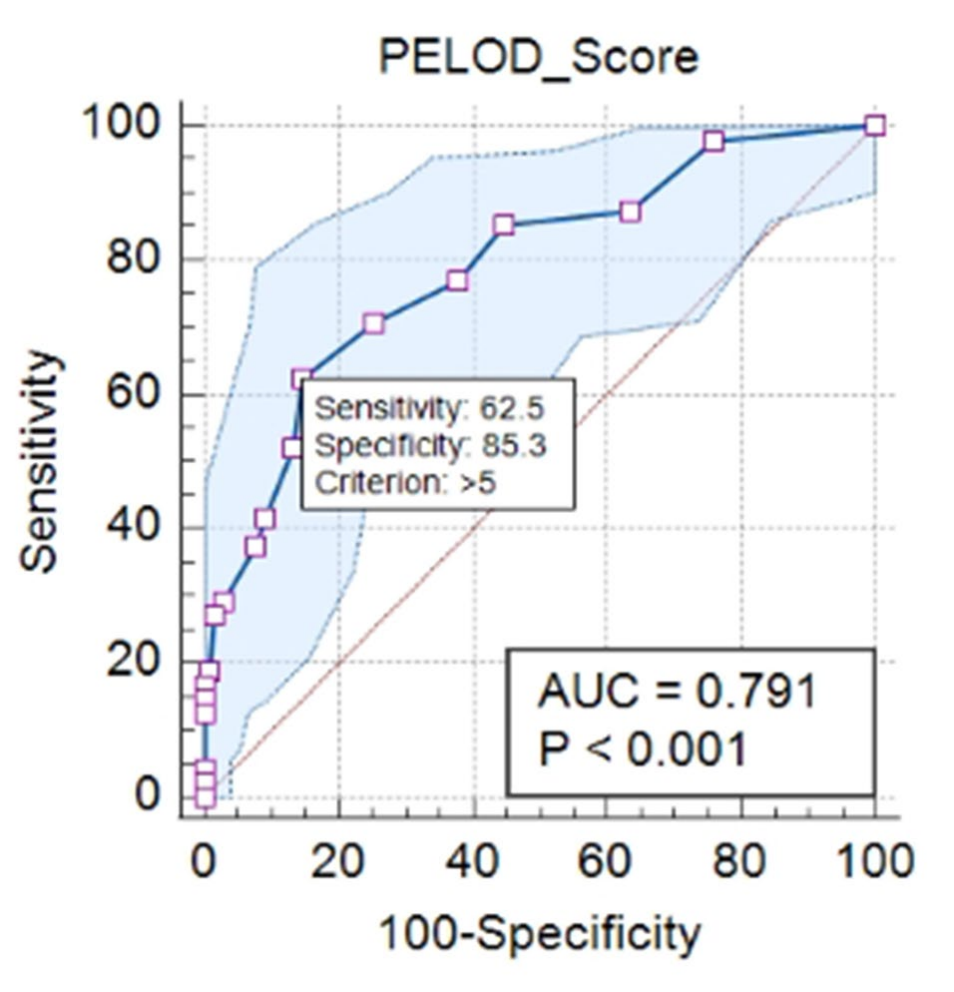
PELOD 2-Score ROC

We were able to calculate the PELOD 2-score for 177 patients admitted to PICU. The mean (± SD) PELOD 2- score was 7.7(± 5.1) for the group of patients who died, and 3.0 (± 2.9) for the group who survived. This mean difference is statistically significant with *P* < 0.0001. In this study, The PELOD 2_Score cut-off result was 5, with a sensitivity of 65.5% and a specificity of 85.35% (Fig. 4).

**Fig. 5:**
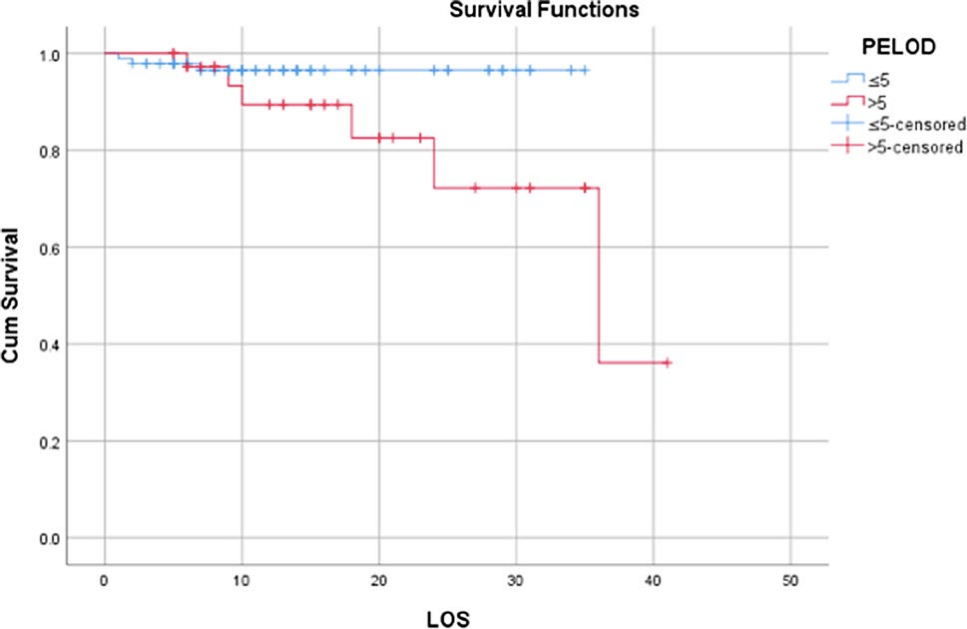
Kaplan Meier

By obtaining this cut off point value, we divided the subjects based on their PELOD 2-score ≤ 5 and PELOD 2 –score > 5 and analyzed it with Kaplan Meier curve (Fig. 5). The curve reveals that patients with a PELOD 2-Score of ≤ 5 demonstrate higher survival compared to those with a PELOD 2-Score > 5.

## Discussion

We found that only 59.7% of all patient with critically ill referral cases who arrived in our hospital had a true indication for PICU admission. This means that over 40% of the patients were in a stable condition and thus considered appropriate for hospitalization in the general ward. This could either be caused by the effectiveness of the initial treatment administered at the referring hospital, or it suggests discrepancies in the assessment of PICU admission indication in pediatric patients between the referring hospital and the referral hospital. Yet, this also contributed a burden of the PICU limited capacity since a PICU bed was unjustified reserved for a substantial number of patients. Multiple studies have identified strategies to address this discrepancies, including enhancing inter-hospital communication through direct interaction with the PICU personnel, enhancing referral documentation, and developing transport system involving the transport team from the referral hospital [18–21].

On the other hand, mortality of the transferred patients was almost 30% (of who more than one-third diedwithin the first 48 h), which is substantial higher than the overall mortality rate of 23.5% in our unit. The death rates reported in the literature is lower than we found in our study and range from 3.9 to 17% [18–20, 22]. Yet, this is influenced by a variety of factors, including demographic and socioeconomic characteristics, disease-related factors, health system-based factors, as well as factors related to pre-referral management, referral processes, and transportation [9, 23–25]. A study conducted by Shinozaki et al., (2021) in Los Angeles, USA, examined the impact of transport time on the mortality rate and length of stay for critically ill pediatric patients undergoing inter-facility transport [18]. The findings of this study indicated that there was no significant association between the duration of transport time and these outcomes. Other study done by Seaton et al., (2020) in United Kingdom found transport time to PICU of referral hospital may be associated with a small reduction in PICU length of stay [20]. Both studies were conducted within the setting of developed countries, where the availability of transport teams for critical ill children was present.

Taken together, our findings imply that there is room for improvement of the initial treatment and stabilization of the patients in the referring hospitals as well as improvement of the transportation system. Indeed in many studies it has been reported that the lack of organized emergency and intensive care services and expertise in the peripheral hospitals, causes referral hospitals to bear large numbers of referrals and admissions particularly those with unfavorable outcomes [8, 9, 26].

In our study, we observed disparities in PICU admission indication between the referring hospital and the referral hospital, particularly with the favorable patient state upon arrival at our ED. The most common diagnosis to refer patients to PICU was shock, respiratory distress/failure, or CNS dysfunction. In line with aforementioned discrepancies a disparity exists between the emergency diagnosis rendered by the referring hospital and the emergency diagnostic rendered at the referral hospital.

There are several possible options for addressing these problems include enhancing transportation infrastructure, providing comprehensive training for doctors in peripheral hospitals, establishing standardized protocols for pediatric emergency assessments, and implementing telemedicine consultations [27–30]. These opportunities have the potential to contribute to the improvement of the overall referral process for critically ill children. More studies must be done to investigate those problems for the purpose to improve the overall effectiveness of the referral system for pediatric critical ill patients. It is expected that improving the referral system for critically ill children will contribute to a reduction in child mortality in our setting.

With the objective to capture the clinical profile and outcome of critically ill children referred to a tertiary hospital our study has several limitations. This study had been done in only one tertiary referral hospital in Indonesia and in a short time period, there may be difference characteristics found in other referral hospitals with longer study duration. Additionally, the findings may not fully represent different healthcare settings or regions, and establishing causality remains challenging. We also lacked sufficient data on the therapy conducted before referral and the specific stabilization efforts implemented.

However, this is among the first studies to investigate the referral system of critically ill child in Indonesia. Further investigation is necessary to examine the concerns surrounding the referral system in critically ill children, particularly for determining the reasons for delayed response and transfer time, with the goal of improving patient survival.

## Conclusion

Shock is the most common pre-referral emergency diagnosis. High mortality rates, discrepancy in PICU admission indication and a long referral process are the problems surrounding pediatric critical ill referral case that we encounter in our setting.

## Data Availability

All data generated or analyzed during this study are included in the submission. The raw data are available from the corresponding author on reasonable request.

## Abbreviations

PICU: Pediatric intensive care unit
PELOD: Pediatric Logistic Organ Dysfunction
HR: Hazard ratio
